# Promiscuous activities of heterologous enzymes lead to unintended metabolic rerouting in *Saccharomyces cerevisiae* engineered to assimilate various sugars from renewable biomass

**DOI:** 10.1186/s13068-018-1135-7

**Published:** 2018-05-14

**Authors:** Eun Ju Yun, Eun Joong Oh, Jing-Jing Liu, Sora Yu, Dong Hyun Kim, Suryang Kwak, Kyoung Heon Kim, Yong-Su Jin

**Affiliations:** 1 Carl R. Woese Institute for Genomic Biology, University of Illinois at Urbana-Champaign, Urbana, IL 61801 USA; 2 Department of Biotechnology, Graduate School, Korea University, Seoul, 02841 South Korea

**Keywords:** Metabolite profiling, *Saccharomyces cerevisiae*, Renewable biomass, Carbon source, Promiscuous activity, Autophagy

## Abstract

**Background:**

Understanding the global metabolic network, significantly perturbed upon promiscuous activities of foreign enzymes and different carbon sources, is crucial for systematic optimization of metabolic engineering of yeast *Saccharomyces cerevisiae*. Here, we studied the effects of promiscuous activities of overexpressed enzymes encoded by foreign genes on rerouting of metabolic fluxes of an engineered yeast capable of assimilating sugars from renewable biomass by profiling intracellular and extracellular metabolites.

**Results:**

Unbiased metabolite profiling of the engineered *S. cerevisiae* strain EJ4 revealed promiscuous enzymatic activities of xylose reductase and xylitol dehydrogenase on galactose and galactitol, respectively, resulting in accumulation of galactitol and tagatose during galactose fermentation. Moreover, during glucose fermentation, a trisaccharide consisting of glucose accumulated outside of the cells probably owing to the promiscuous and transglycosylation activity of β-glucosidase expressed for hydrolyzing cellobiose. Meanwhile, higher accumulation of fatty acids and secondary metabolites was observed during xylose and cellobiose fermentations, respectively.

**Conclusions:**

The heterologous enzymes functionally expressed in *S. cerevisiae* showed promiscuous activities that led to unintended metabolic rerouting in strain EJ4. Such metabolic rerouting could result in a low yield and productivity of a final product due to the formation of unexpected metabolites. Furthermore, the global metabolic network can be significantly regulated by carbon sources, thus yielding different patterns of metabolite production. This metabolomic study can provide useful information for yeast strain improvement and systematic optimization of yeast metabolism to manufacture bio-based products.

**Electronic supplementary material:**

The online version of this article (10.1186/s13068-018-1135-7) contains supplementary material, which is available to authorized users.

## Background

The yeast *Saccharomyces cerevisiae* has been widely used as an industrial workhorse for manufacture of bio-based products including fuels and other chemicals owing to its advantages over bacteria such as the zero risk of phage infection, higher tolerance to environmental stress, and genetic traceability [1, 2]. To achieve a cost-effective biorefinery process via *S. cerevisiae*, its capability of efficient utilization of diverse substrates from various types of biomass is a prerequisite [3]. For efficient utilization of various sugars generated from biomass feedstocks including lignocellulose by *S. cerevisiae*, strain improvement, enabling the assimilation of the sugars that are naturally non-fermentable by *S. cerevisiae* such as xylose [4] and cellobiose [5] and the simultaneous fermentation of mixed sugars, have been attempted on many occasions by means of genetic perturbations [5, 6].

In our previous studies, *S. cerevisiae* has been successfully engineered to catabolize xylose and cellobiose simultaneously (resulting in strain EJ4) using multiple genetic perturbations and rational and laboratory evolution, as follows [7]. Initially, the oxidoreductive xylose-assimilating pathway consisting of *XYL1*, *XYL2*, and *XYL3* coding for xylose reductase (XR), xylitol dehydrogenase (XDH), and xylulokinase, respectively, from *Scheffersomyces stipitis* [8] was introduced into *S. cerevisiae* D452-2 by multicopy integration (resulting in strain SR7) [9]. Then, strain SR8, a fast and efficient xylose-fermenting strain, was derived from strain SR7 through serial subculturing of strain SR7 in the presence of xylose followed by deletion of *ALD6* coding for acetaldehyde dehydrogenase to minimize acetate production [9]. Next, the cellobiose-assimilating pathway consisting of *cdt*-*1* and *gh1*-*1* coding for cellodextrin transporter and β-glucosidase, respectively, from fungus *Neurospora crassa* was introduced into strain SR8 (resulting in strain EJ1) [7, 10]. Through serial subculturing of strain EJ1 in the presence of cellobiose, strain EJ4 with efficient and simultaneous fermenting capability for xylose and cellobiose was finally created [7].

Recent strategies for stain improvement have been focused more on the identification of genes or metabolites involved in the regulation of the metabolic pathways associated with target substrates or products via comprehensive ‘omics’ approaches [11–13]. For example, genome sequencing results on a yeast strain evolved to ferment xylose rapidly revealed a non-synonymous single nucleotide polymorphism in *PHO13* coding for haloacid dehalogenase type IIA phosphatase [9]. By transcriptome analysis, it was revealed that the mutation in *PHO13* upregulates the pentose phosphate pathway, a major catabolic pathway for xylose, in yeast [14, 15].

To investigate metabolism and phenotypic status in living organisms, metabolomics is a promising tool [16]. For instance, metabolomics has been applied to yeast strain improvement by measurement of intracellular metabolic flux or the redox balance [17]. In particular, when engineered *S. cerevisiae* grows on xylose, increased metabolic flux in the tricarboxylic acid (TCA) cycle and an imbalance between NADH and NADPH were revealed by a metabolic flux analysis based on the ^13^C-labelled metabolite analysis by gas chromatography/mass spectrometry (GC/MS) [18]. Recent studies using metabolomics have led to the understanding of the stress response caused by fermentation inhibitors generated during pretreatment of biomass and to increase stress tolerance [17]. For instance, by quantitative metabolite analysis, proline and *myo*-inositol were found to be the key metabolites for tolerance of *S. cerevisiae* against furfural, acetic acid, and phenol [19]. Besides, metabolomics-based regression modelling has been employed to identify threonine as the key metabolite for tolerance of *S. cerevisiae* against 1-butanol [20]. Through this metabolomic approach, *S. cerevisiae* exhibiting enhanced tolerance against 1-butanol was successfully constructed by deleting the genes associated with threonine metabolism [20]. Nonetheless, metabolomic elucidation of global metabolic changes in *S. cerevisiae* in response to various carbon sources derived from lignocellulosic or marine macroalgal biomass has not been performed yet.

In this study, we aimed to investigate the effects of promiscuous activities of enzymes encoded by foreign genes and various carbon sources from renewable biomass on global metabolic networks in engineered yeast, which have been usually overlooked in metabolic engineering of yeast. To accomplish this, intracellular and extracellular metabolites of strain EJ4 grown on glucose, cellobiose, galactose, or xylose were analyzed by GC/MS, and comprehensively compared by principal component analysis (PCA) and hierarchical cluster analysis (HCA). These metabolomic analytical results may provide useful information for systematic optimization of yeast metabolism to effectively manufacture bio-based products from renewable biomass.

## Results

### Cell growth of strain EJ4 on various carbon sources

**Fig. 1:**
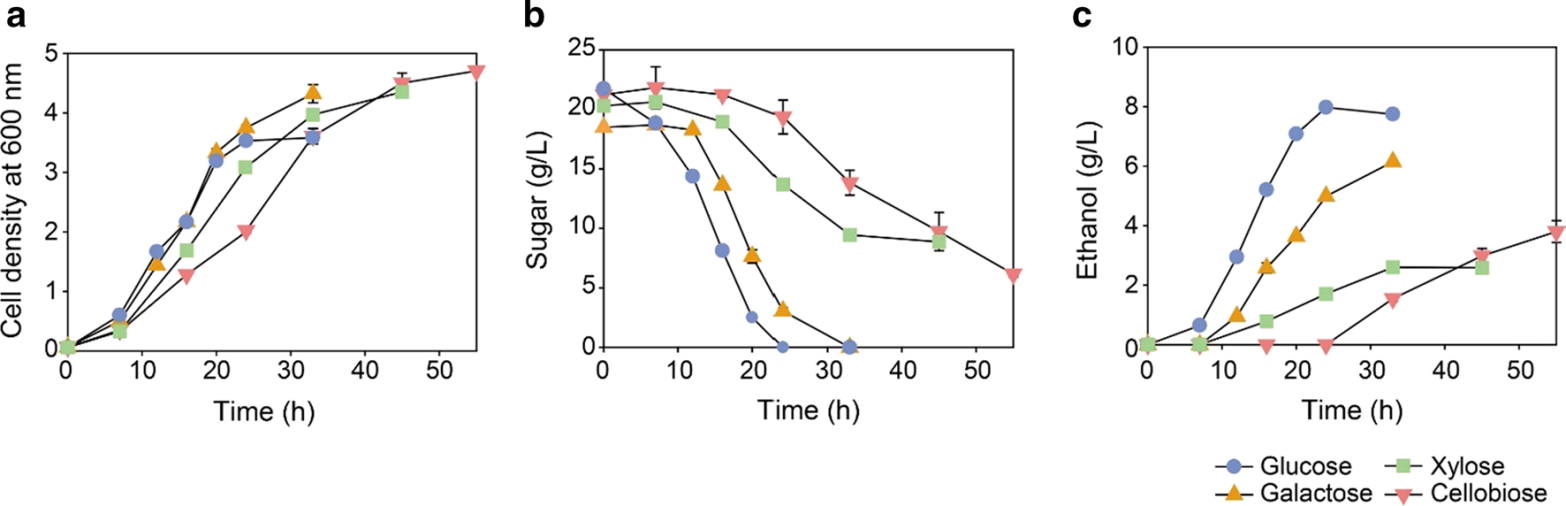
Comparison of the fermentation profiles of *S. cerevisiae* strain EJ4 grown on different carbon sources. **a** Cell growth, **b** sugar consumption, and **c** ethanol production of strain EJ4 during the fermentation of glucose, galactose, xylose, or cellobiose. Strain EJ4 was cultivated at 30 °C and 200 rpm in yeast synthetic complete broth containing 6.7 g/L yeast nitrogen base, 0.79 g/L complete supplement mixture, and 20 g/L one of carbon sources

Cell growth, sugar consumption, and ethanol production of strain EJ4 differed significantly when four different sugars (glucose, galactose, xylose, or cellobiose) were provided as a carbon source (Fig. 1). Among the four sugars, the higher growth rates were observed when glucose or galactose was used as a carbon source (Fig. 1a). Meanwhile, the lowest growth rate was observed during the growth on cellobiose (Fig. 1a). During the xylose fermentation by strain EJ4, xylose consumption ceased after 33 h (Fig. 1b). For this reason, the lowest ethanol production was achieved from xylose (Fig. 1c). The highest ethanol production was achieved from glucose (Fig. 1c).

### HCA of the intracellular metabolites of strain EJ4 grown on different carbon sources

For comparing the intracellular metabolite profiles of strain EJ4 grown on the four different sugars (glucose, galactose, xylose, or cellobiose), sampling of culture broth was performed twice for each sugar in the exponential phase of growth as follows: glucose, at 12 and 16 h; galactose, at 16 and 20 h; xylose, at 24 and 33 h; and cellobiose, at 33 and 45 h (Fig. 1a, b). In total, 73 intracellular metabolites including 20 amino acids, 12 sugars, 15 organic acids, 5 fatty acids, 9 phosphates, and 2 nucleosides were identified in strain EJ4 (Additional file 1: Table S1).

**Fig. 2:**
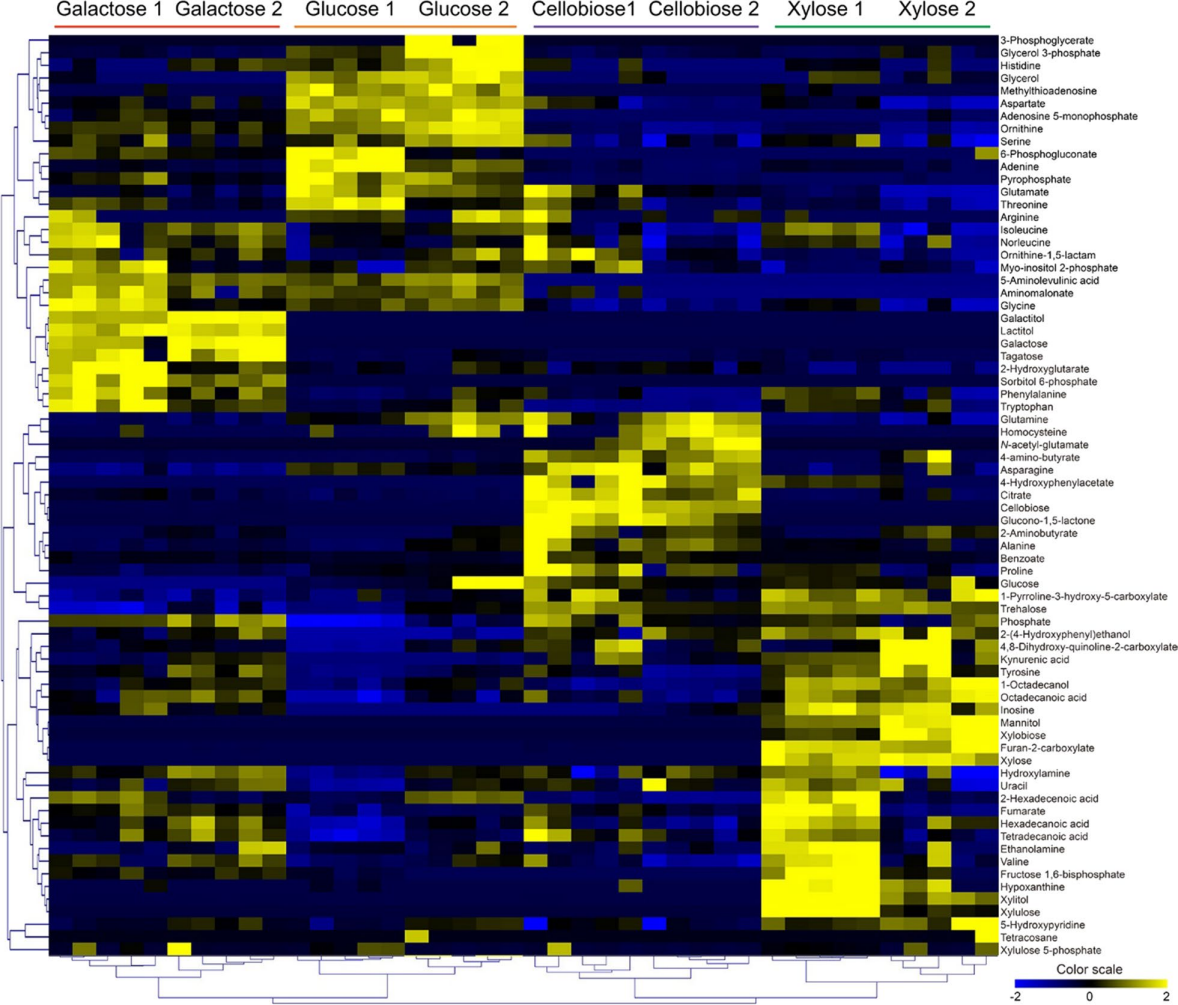
A heat map of 73 intracellular metabolites of *S. cerevisiae* strain EJ4 grown on four different carbon sources, glucose, galactose, xylose, or cellobiose, until the exponential growth phase. The *x*-axis labels represent the four different carbon sources with two different sampling timepoints, 1, mid-exponential phase; 2, late-exponential phase. All experiments were performed in five replicates

The identified metabolites from a total of 40 samples (4 carbon sources × 2 sampling timepoints × 5 replicates) were subjected to HCA to identify possible differences in the intracellular metabolite profiles of strain EJ4 grown on glucose, galactose, xylose, or cellobiose (Fig. 2). Clustering of the intracellular metabolites of strain EJ4 resulted in good separation based on the type of consumed sugars (Fig. 2). In particular, increased levels of sugars and sugar derivatives including galactose, tagatose, galactitol, lactitol, and sorbitol 6-phosphate were observed during the growth on galactose (Fig. 2). Meanwhile, increased levels of the metabolites associated with the lower chain of glycolysis such as 3-phosphoglycerate, glycerol 3-phosphate, and glycerol and some amino acids such as histidine, aspartate, ornithine, serine, adenine, glutamine, and threonine were observed during the growth on glucose (Fig. 2). In addition, when cellobiose was used as a carbon source, four amino acids such as glutamine, homocysteine, asparagine, and proline were abundantly produced by strain EJ4 (Fig. 2). Especially, during the growth on xylose, remarkable abundances of most of the fatty acids identified in this study were observed such as 1-octadecanol, octadecanoic acid, hexadecanoic acid, 2-hexadecenoic acid, and tetradecanoic acid (Fig. 2). Moreover, higher levels of the metabolic intermediates associated with the pentose phosphate (PP) pathway such as xylose, xylitol, xylulose, and xylulose 5-phosphate were observed during the growth on xylose (Fig. 2).

### PCA of the intracellular metabolites of strain EJ4 grown on different carbon sources

**Fig. 3:**
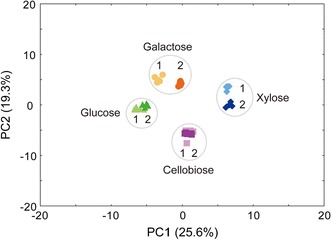
A PCA scatter plot generated from the 73 intracellular metabolites of *S. cerevisiae* strain EJ4 grown on four different carbon sources, glucose, galactose, xylose, or cellobiose, until the exponential growth phase. The labels in the PCA scatter plot represent the four different carbon sources with two different sampling timepoints, 1, mid-exponential phase; 2, late-exponential phase. All experiments were performed in five replicates

To explore any intrinsic similarities and differences in the intracellular metabolite profiles of the EJ4 cells grown on different sugars, PCA was performed. Similar to the results obtained in HCA (Fig. 2), two-dimensional PCA results uncovered clear separation among the 4 carbon sources, with an *R*^2^*X* value of 0.449, which indicates goodness of fit of the PCA model (Fig. 3). To be precise, PC1 primarily contributed to the separation of the metabolite profiles obtained when glucose or xylose was used as a carbon source (Fig. 3). Meanwhile, PC2 contributed to the separation of the metabolite profiles obtained when galactose or cellobiose was used as a carbon source (Fig. 3).

The loading scores for the top 20 metabolites that contributed primarily to PC1 and PC2 are listed in Additional file 2: Table S2. Sugars and sugar alcohols such as xylose, trehalose, xylitol, and mannitol were the major metabolites that contributed to PC1 the most positively. Meanwhile, non-proteinogenic amino acids such as ornithine and 5-aminolevulinic acid, and proteinogenic amino acids such as aspartate, threonine, and glutamine contributed to PC1 the most negatively (Additional file 2: Table S2). Furthermore, aromatic amino acids such as tryptophan and phenylalanine and sugars and sugar derivatives such as galactose, tagatose, lactitol, galactitol, and sorbitol 6-phosphate contributed to PC2 the most positively. Meanwhile, asparagine, cellobiose, glucono-1,5-lactone, citrate, and glutamine were the top five metabolites that contributed to PC2 the most negatively (Additional file 2: Table S2).

### Notable metabolites in the EJ4 cells growing on galactose

**Fig. 4:**
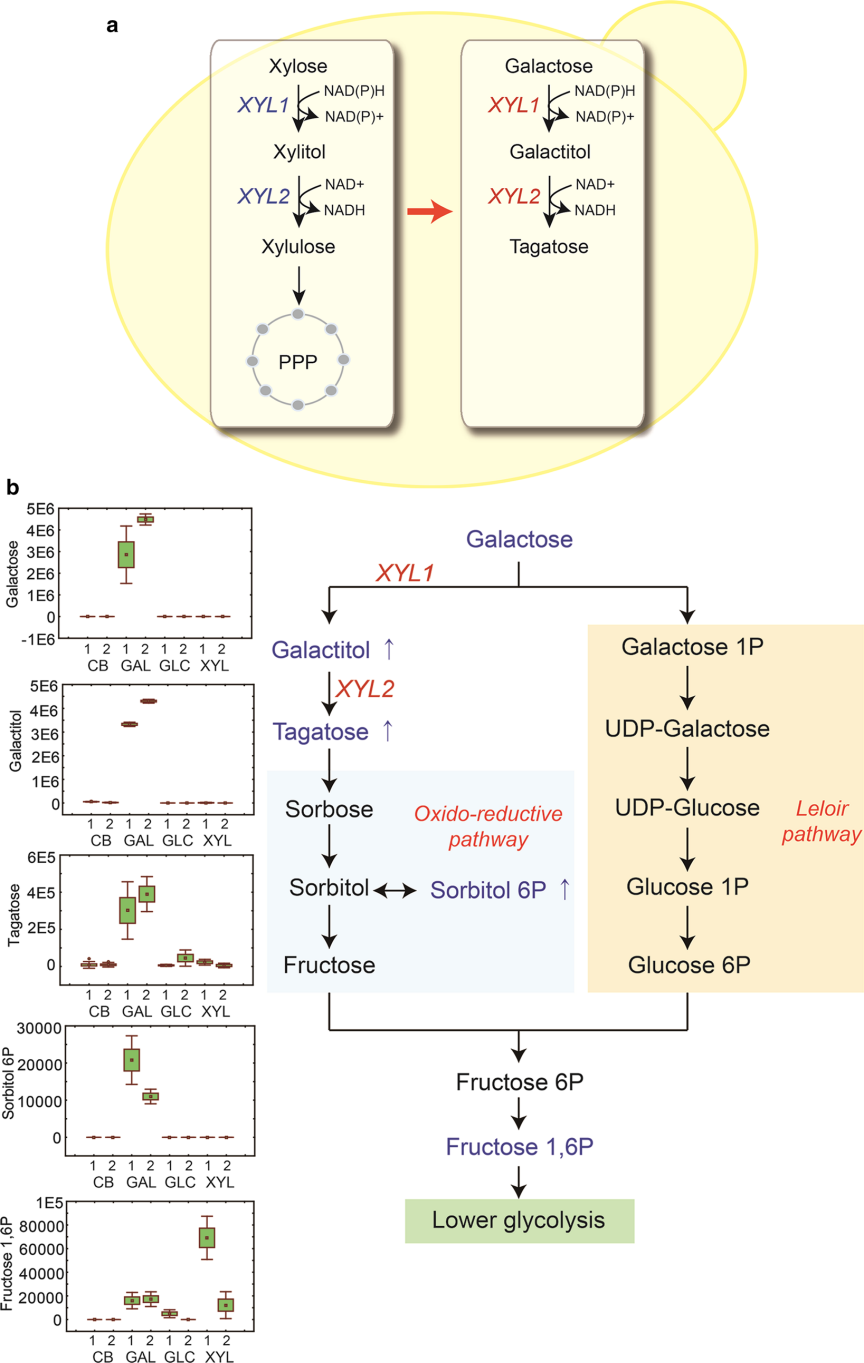
**a** A schematic diagram presenting the promiscuous activities of xylose reductase (encoded by *XYL1*) and xylitol dehydrogenase (encoded by *XYL2*) on galactose and galactitol, respectively. **b** The oxidoreductive pathway and Leloir pathway for galactose catabolism. Normalized abundance levels of the intracellular metabolites of *S. cerevisiae* strain EJ4 grown on galactose (GAL), glucose (GLC), xylose (XYL), or cellobiose (CB) related to the galactose catabolism are shown in the box-whisker plots. The *x*-axis labels in the box-whisker plots represent the four different carbon sources with two different sampling timepoints, 1, mid-exponential phase; 2, late-exponential phase

**Fig. 5:**
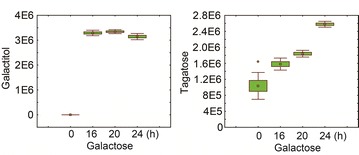
Extracellular accumulation of galactitol and tagatose presented in the box-whisker plots during galactose fermentation by strain EJ4

According to the PCA and HCA analyses described above, we examined relative abundance changes in intracellular and extracellular metabolites present in the EJ4 cells growing on different carbon sources. When strain EJ4 was grown on galactose, significant increases in galactitol and d-tagatose abundances were observed probably due to the promiscuous activities of XR and XDH encoded by *XYL1* and *XYL2*, respectively (Fig. 4a, b). Enzyme promiscuity is the capability of an enzyme to catalyze alternative reactions other than the native reaction [21, 22]. Moreover, a higher level of sorbitol 6-phosphate, a metabolic intermediate associated with the oxidoreductive pathway of galactose, was detected during the growth on galactose (Fig. 4b). Consistent with the intracellular metabolite profiles, higher extracellular accumulation of galactitol and d-tagatose was also observed during the growth on galactose (Fig. 5). When galactose was depleted after 33 h, the final concentrations of galactitol and tagatose were 0.40 and 0.31 g/L, respectively (Additional file 3: Figure S1).

### Notable metabolites in the EJ4 cells growing on glucose

**Fig. 6:**
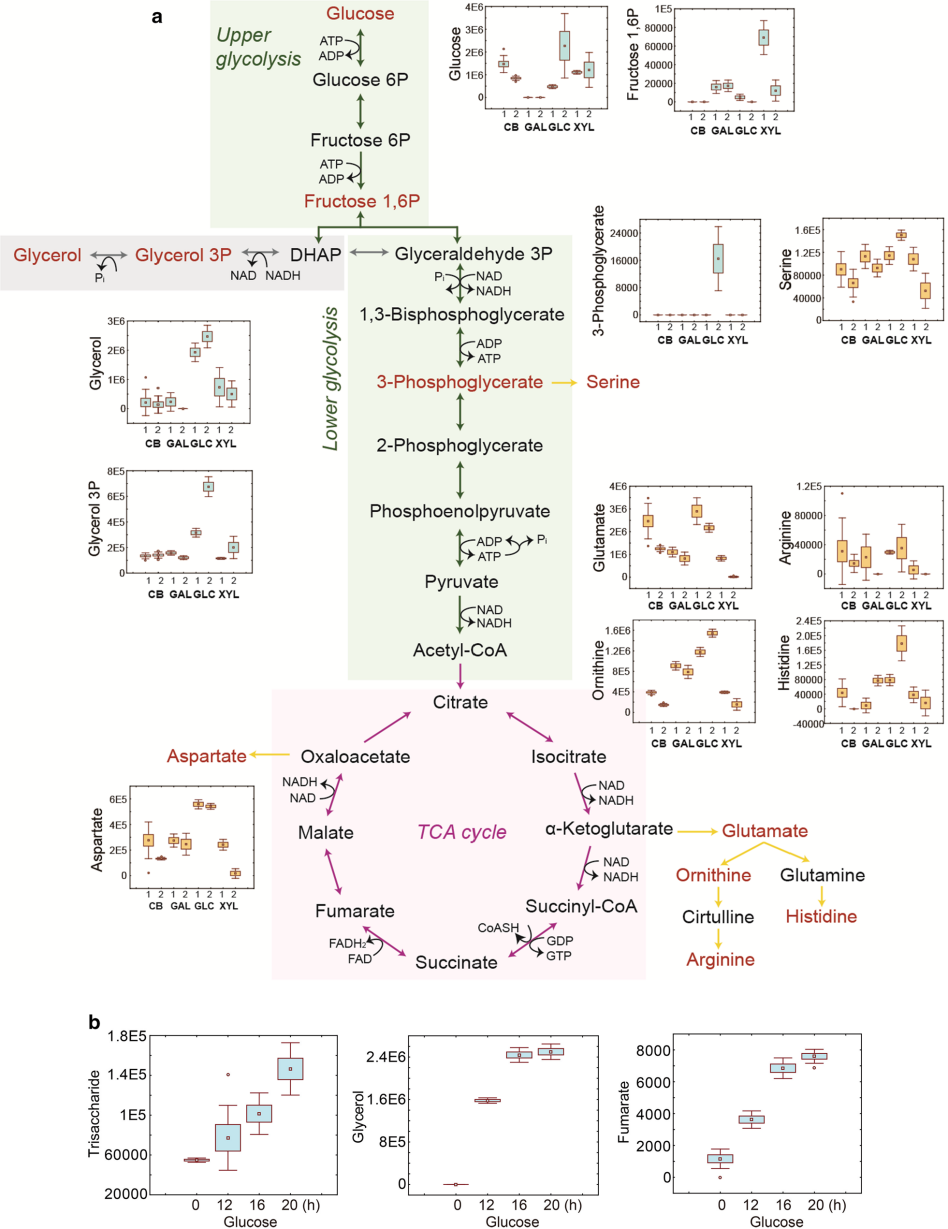
**a** The most common type of glycolysis, the Embden–Meyerhof–Parnas (EMP) pathway, and the TCA cycle. The normalized abundance levels of the intracellular metabolites of *S. cerevisiae* strain EJ4 grown on galactose (GAL), glucose (GLC), xylose (XYL), or cellobiose (CB) related to the EMP pathway and TCA cycle are shown in box-whisker plots. The *x*-axis labels in the box-whisker plots represent the four different carbon sources with two different sampling timepoints, 1, mid-exponential phase; 2, late-exponential phase. **b** Extracellular accumulation of trisaccharide, glycerol, and fumarate during glucose fermentation by strain EJ4 is presented in box-whisker plots

When cells are growing on glucose, higher abundances of an intermediate from the lower chain of glycolysis, 3-phosphoglycerate, metabolites derived from the lower chain of glycolysis such as glycerol and glycerol 3-phosphate, and amino acids derived from the TCA cycle such as glutamine, ornithine, histidine, and aspartate were detected (Fig. 6a). During time-course monitoring of extracellular metabolites, the abundances of glycerol and fumarate increased (Fig. 6b). Interestingly, a trisaccharide consisting of glucose was also secreted during glucose fermentation (Fig. 6b).

### Notable metabolites in the EJ4 cells growing on xylose

**Fig. 7:**
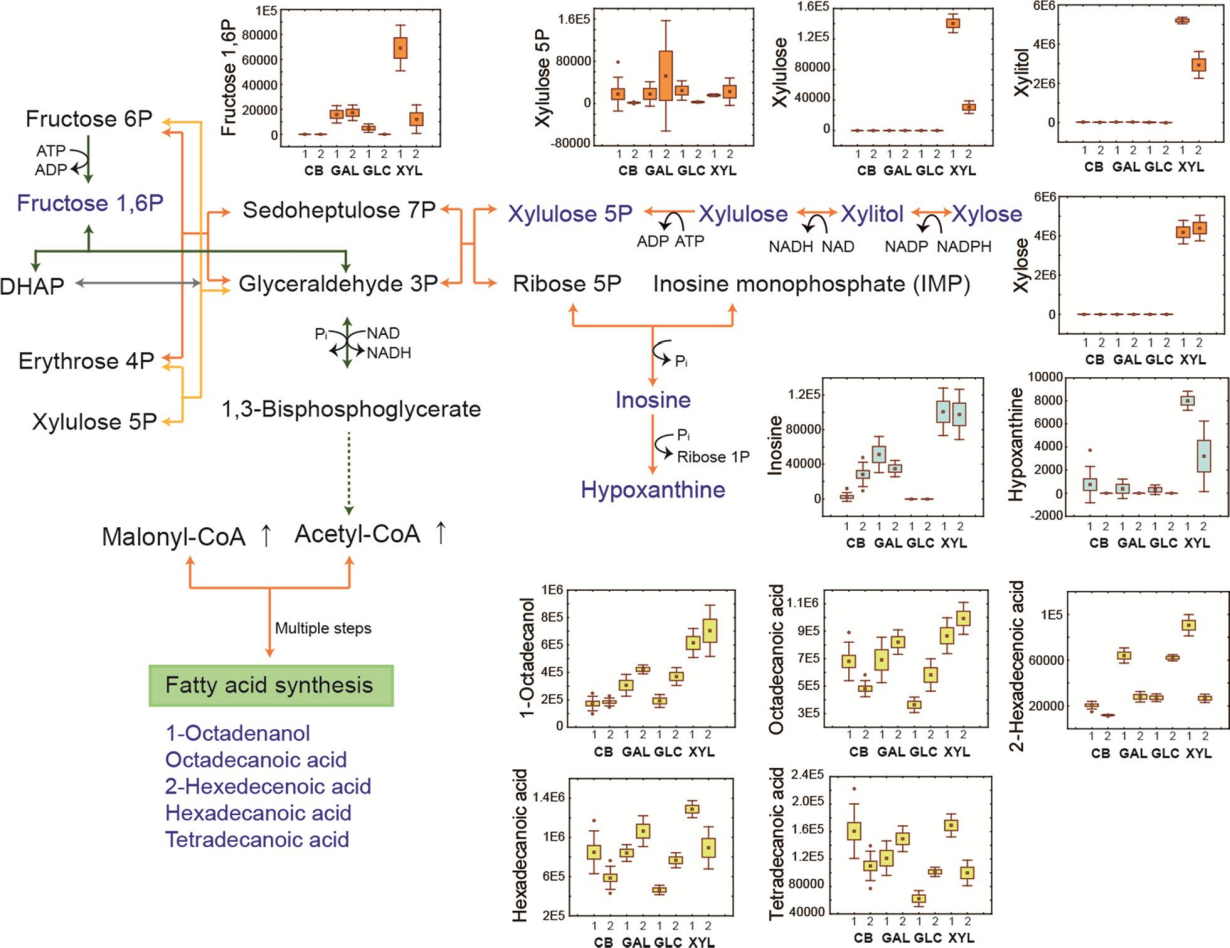
The PP pathway and fatty acid biosynthesis. Normalized abundance levels of the intracellular metabolites of *S. cerevisiae* strain EJ4 grown on galactose (GAL), glucose (GLC), xylose (XYL), or cellobiose (CB) related to the PP pathway and fatty acid biosynthesis are shown in box-whisker plots. The *x*-axis labels in the box-whisker plots represent the four different carbon sources with two different sampling timepoints, 1, mid-exponential phase; 2, late-exponential phase

The intracellular metabolite profiles of strain EJ4 growing on xylose revealed that the metabolic intermediates associated with the PP pathway, the major catabolic pathway of xylose, such as xylose, xylitol, and xylulose were highly abundant during the growth on xylose (Fig. 7). Additionally, higher levels of metabolic intermediates derived from the PP pathway such as inosine and hypoxanthine were observed (Fig. 7). Meanwhile, most of the fatty acids identified in this study, such as 1-octadecanol, octadecanoic acid, hexadecanoic acid, and 2-hexadecenoic acid, were also highly produced in the cells growing on xylose (Fig. 7). Among the fatty acids abundantly produced under xylose, the abundances of hexadecenoic and 2-hexadecenoic acid were lower in the late-exponential phase than those in the mid-exponential phase (Fig. 7).

### Notable metabolites in the EJ4 cells growing on cellobiose

**Fig. 8:**
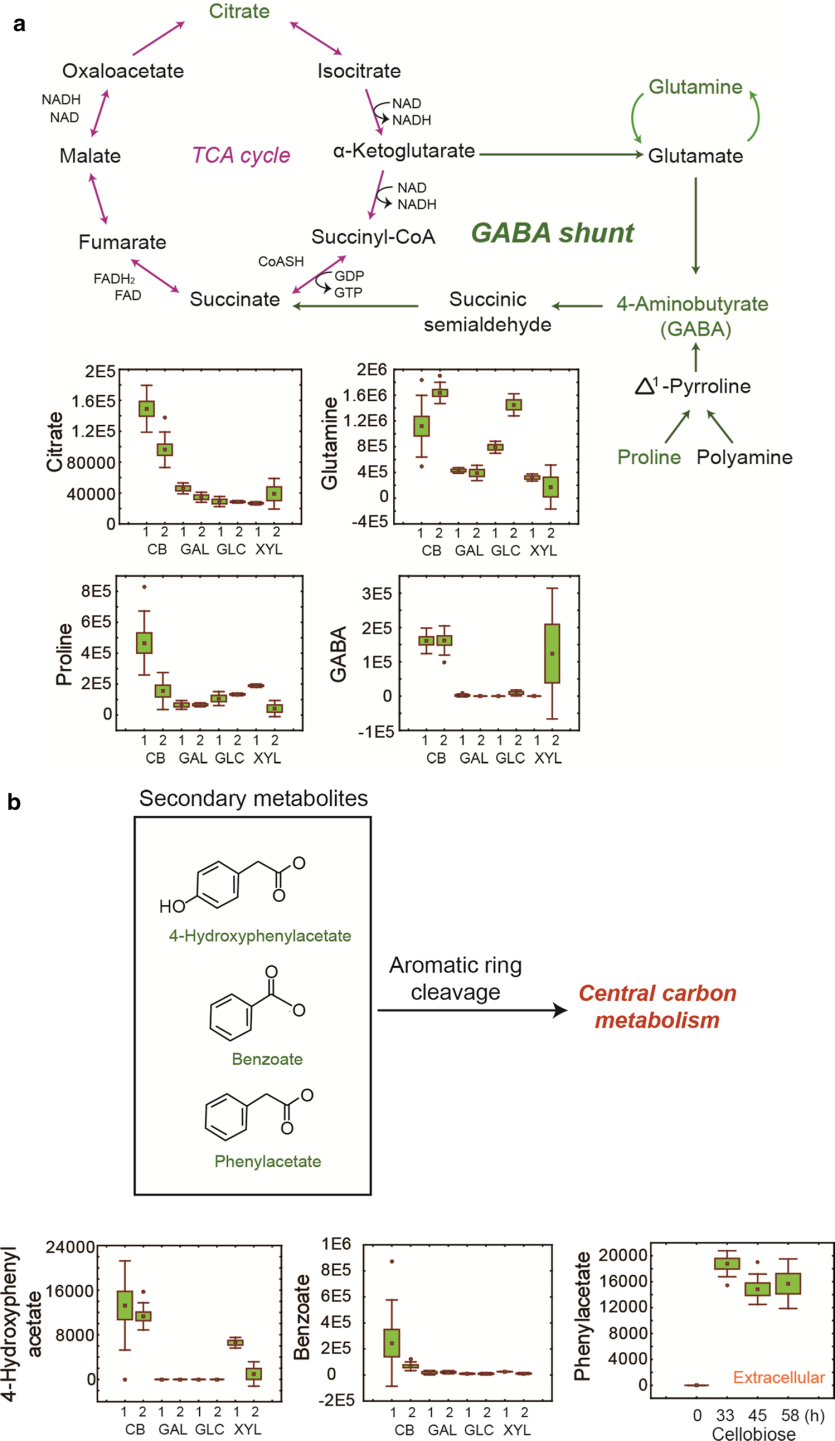
**a** The TCA cycle and GABA shunt pathway. Normalized abundance levels of the intracellular metabolites of *S. cerevisiae* strain EJ4 grown on galactose (GAL), glucose (GLC), xylose (XYL), or cellobiose (CB) related to the TCA cycle and GABA shunt pathway are shown in box-whisker plots. The *x*-axis labels in the box-whisker plots represent the four different carbon sources with two different sampling timepoints, 1, mid-exponential phase; 2, late-exponential phase. **b** Intracellular and extracellular accumulation of secondary metabolites, 4-hydroxyphenylacetate, benzoate, and phenylacetate, during cellobiose fermentation by strain EJ4 is presented in box-whisker plots

When grown on cellobiose, increased abundance of citrate, an intermediate of the TCA cycle, was observed in the intracellular metabolite profiles of strain EJ4 (Fig. 8a). Moreover, higher levels of the metabolic intermediates of the γ-aminobutyric acid (GABA) shunt pathway, including GABA, glutamine, and proline, were observed (Fig. 8a). In particular, secondary metabolites such as phenolic compounds including 4-hydroxyphenylacetate and benzoate were abundantly produced by strain EJ4 growing on cellobiose (Fig. 8b). As in the intracellular metabolite profile, a higher level of phenylacetate was detected in the culture media during cellobiose fermentation (Fig. 8b).

## Discussion

In this study, we aimed to delve into metabolic phenotypes of *S. cerevisiae* strain EJ4 (that was engineered to simultaneoulsy catabolize xylose and cellobiose) using various carbon sources from renewable biomass including lignocellulose and red macroalgae. To this end, intracellular and extracellular metabolites of strain EJ4 grown on glucose, galactose, xylose, or cellobiose were comparatively profiled. As expected, different metabolic patterns were identified depending on the carbon sources added to the culture media. However, promiscuous activities of the heterologous enzymes, the coding genes of which were present and functionally expressed originally to catabolize xylose and cellobiose in strain EJ4, were unexpectedly observed from our intracellular and extracellular metabolite analysis. The promiscuous activities were found to lead to metabolic rerouting which could result in a low yield and productivity of a desired product due to the unintended formation of other  metabolites. This is the first finding of promiscuous activities of heterologous enzymes in metabolically engineered microorganisms.

Enzyme promiscuity indicates the capability of catalyzing alternative reactions in addition to its native catalysis reaction [21, 22]. Enzyme promiscuity appears to catalyze distinctly different chemical reactions (i.e., catalytic promiscuity) or to show broad substrate specificity (i.e., substrate promiscuity) [22, 23]. After heterologous genes are introduced into an engineered microbial host, promiscuous activities of heterologous enzymes can lead to undesirable by-products accumulation via alternative catalytic routes. However, promiscuous enzyme activities of heterologous genes are often overlooked in the process of optimizing engineered strains. In strain EJ4 assimilating xylose and cellobiose in this study, promiscuous activities of heterologous enzymes were shown by XR and XDH encoded by *XYL1* and *XYL2* which were introduced to catabolize xylose. When EJ4 was grown on galactose, promiscuous activities of XR and XDH caused accumulation of galactitol and tagatose, respectively, in strain EJ4 (Figs. 4b, 5). The lower yield and productivity of ethanol during galactose fermentation than during glucose fermentation (Fig. 1c) was probably due to the significant accumulation of galactitol and d-tagatose. For assimilation of xylose, there exist two major pathways: the oxidoreductive pathway composed of XR and XDH, mainly found in fungi such as *S. stipitis* [8] and the isomerase pathway composed of xylose isomerase, mainly found in bacteria such as *Clostridium phytofermentans* [24] and anaerobic fungi [25]. In particular, XR (EC. 1.1.1.21) has broad substrate specificity and is known to be involved in other fungal sugar metabolic pathways. For example, in *Hypocrea jecorina*, XR is induced by various substrates including d-xylose, l-arabinose, lactose, and l-galactose [26]. In fungi such as *H. jecorina* and *Aspergillus niger*, the oxidoreductive galactose pathway is initiated with reduction of d-galactose to galactitol by XR encoded by *XYL1*, and galactitol is then converted to l-xylo-3-hexulose by galactitol dehydrogenase acting on C4 [23, 24]. In the present study, however, d-tagatose was produced instead of l-xylo-3-hexulose because XDH encoded by *XYL2* acts on C2 [27]. d-tagatose production by XDH was demonstrated here by fermentation of galactose by strain EJ4 (Figs. 4b, 5) and by an in vitro activity assay of the crude enzymes of EJ4 by using galactitol as a substrate (Additional file 4: Figure S2). To our knowledge, this is the first report of the galactitol dehydrogenase activity of XDH encoded by *XYL2* acting on galactitol to produce d-tagatose. Furthermore, sorbitol 6-phosphate, a metabolic intermediate associated with the oxidoreductive galactose pathway, was detected only during the growth on galactose (Fig. 4b). Thus, the new alternative galactose catabolic reactions catalyzed by XR and XDH may be linked to the oxidoreductive galactose pathway (Fig. 4b).

In addition to xylose, strain EJ4 was engineered to assimilate cellobiose. During the fermentation of glucose by strain EJ4, extracellular accumulation of a trisaccharide consisting of glucose was observed in this study. It was probably because of a promiscuous activity of the β-glucosidase encoded by *gh1*-*1* originating from fungus *N. crassa*, which is the transglycosylation activity (Fig. 6b and Additional file 5: Figure S3). The transglycosylation activity of β-glucosidase may have resulted in producing a trisaccharide by adding a glucose moiety to the disaccharide, cellobiose. Introduction of a cellobiose-assimilating pathway consisting of *cdt*-1 and *gh1*-*1* into *S. cerevisiae* enables simultaneous co-fermentation of cellobiose with other sugars such as xylose and galactose by removing the catabolic repression and rigid flux partition toward ethanol production (i.e., the Crabtree effect) caused by glucose [5, 28]. Simultaneous utilization of various substrates from biomass increases the yield and productivity of the final products (e.g., ethanol) and reduces overall fermentation time [7]. Nevertheless, the transglycosylation activity of β-glucosidase can also lower the yield and productivity of a target product [29]. Regarding metabolic fluxes, in the presence of glucose, higher levels of glycerol and glycerol 3-phosphate and a lower level of inorganic phosphate were observed (Fig. 6a, b); these results are probably due to the higher availability of the NADH cofactor [30] and the imbalanced reactions in glycolysis in the presence of ample glucose, respectively [31].

During the growth on cellobiose, increased levels of the metabolic intermediates associated with the GABA shunt pathway were detected (Fig. 8a). In yeast, the GABA shunt pathway, an alternative route for the conversion of α-ketoglutarate to succinate, plays a crucial role in protecting cells from various stressors such as heat [32] and oxidative stress [33]. For example, deletion of the genes related to the GABA shunt pathway in *S. cerevisiae* causes growth defects at a high temperature (i.e., 45 °C) [32]. Furthermore, during the growth on cellobiose, higher levels of secondary metabolites such as 4-hydroxyphenylacetate, benzoate, and phenylacetate were observed (Fig. 8b). Secondary metabolite production usually commences at the late stages of microbial growth such as the stationary or resting phase in response to starvation [34, 35]. On the other hand, a recent study revealed that the plasma membrane ATPase of cellobiose-fermenting yeast is in a carbon starvation-like state, in which the plasma membrane ATPase activates plasma membrane transporters and is closely related to glucose sensing [36]. Therefore, the higher levels of the metabolic intermediates associated with the GABA shunt pathway and secondary metabolite production may be related to the stress responses owing to the carbon starvation-like state of strain EJ4 during cellobiose fermentation (Fig. 8a, b).

Not limited to carbon starvation, autophagy is a cellular response to nutrient deprivation including carbon or nitrogen starvation [37]. As mentioned above, yeast cells growing on cellobiose were in a carbon starvation-like state. In this study, we hypothesized that autophagy would be induced in yeast cells growing on cellobiose. To test this hypothesis, autophagy levels of yeast cells were evaluated in the exponential phase during growth on different carbon sources, glucose, galactose, xylose, and cellobiose, and during glucose depletion (Additional file 6: Figure S4). As expected, higher autophagy levels were observed during glucose depletion than in the presence of glucose because autophagy could be induced by carbon starvation. Moreover, yeast cells grown on cellobiose or xylose showed higher autophagy magnitude, indicating that yeast cells were indeed in a carbon starvation-like state even though both carbon sources were being consumed by yeast cells (Additional file 6: Figure S4).

In addition, we noticed that yeast cells grown on xylose manifested higher fatty acid levels (Fig. 7). This finding is consistent with data from another report showing that starvation is coupled with elevated levels of triglycerides or free fatty acids, which can trigger autophagy [38]. During xylose fermentation, the higher fatty acid production may be related to the higher cytosolic acetyl-CoA level during the growth on xylose than during the growth on other carbon sources (Fig. 7). It has been reported that engineered *S. cerevisiae* grown on xylose shows higher expression of enzymes related to the synthesis of cytosolic acetyl-CoA, the core metabolic intermediate for fatty acid production [39]. Recently, xylose was suggested as a promising carbon source for production of isoprenoids including squalene and amorphadiene, sterol, polyketide, and polyphenol, all of which are synthesised from acetyl-CoA [39, 40].

## Conclusions

In conclusion, the intracellular and extracellular metabolite profiles of the engineered *S. cerevisiae* were found to be significantly affected by carbon sources. Especially, we found promiscuous activities of heterologous enzymes, XR, XDH, and β-glucosidase encoded by *XYL1*, *XYL2*, and *gh1*-*1*, respectively, expressed in strain EJ4. As a result of these promiscuous activities, unexpected metabolites such as galactitol, tagatose, and a trisaccharide of glucose were detected. This is the report of promiscuous activities of heterologous enzymes in metabolically engineered microorganisms. In addition, EJ4 produced the higher levels of fatty acids or secondary metabolites during the growth on xylose or cellobiose, respectively. This metabolomic study can provide useful information for the systematic optimization of yeast metabolic pathways for strain improvement and manufacture of various value-added chemicals using renewable biomass.

## Methods

### Strain and culture conditions

*S. cerevisiae* strain EJ4 capable of efficient xylose and cellobiose fermentation [7] was cultivated in yeast synthetic complete broth containing 6.7 g/L yeast nitrogen base, 0.79 g/L complete supplement mixture (CSM; MP Biomedicals, Solon, OH), and 20 g/L carbon source such as glucose, galactose, xylose, or cellobiose at 30 °C and 200 rpm. Culture samples were collected in the exponential phase of growth for metabolite profiling. Cell growth was monitored by measurement of optical density at 600 nm (OD_600_) using a UV–visible spectrophotometer (Biomate5, Thermo Fisher Scientific, Waltham, MA).

### Preparation of crude enzymes of *S. cerevisiae* strains EJ4 and D452-2

To obtain crude cell-free lysate enzymes of strain EJ4 or the parental strain D452-2, 5 mL of culture that was harvested in the exponential phase of growth was centrifuged at 21,130*×g* and 4 °C for 5 min. The cell pellet was washed by adding 5 mL of phosphate-buffered saline (PBS; pH 7.4) followed by centrifugation at 21,130*×g* and 4 °C for 5 min. The washed cell pellet was resuspended in 1 mL of 20 mM Tris–HCl buffer (pH 7.0). After that, the cells were disrupted by adding 100 µL of glass beads to the suspension and vortexing it for 3 min. Next, soluble cell-free lysate enzymes were obtained by centrifugation at 21,130*×g* and 4 °C for 30 min. Protein concentration was measured using a bicinchoninic acid assay kit (Thermo Fisher Scientific).

### In vitro enzymatic activity assay

To measure galactitol 2-dehydrogenase activity of crude cell-free lysate enzymes of strains EJ4 or D452-2, the reaction mixture containing 1 mg/mL crude cell-free lysate enzymes obtained from EJ4 or D452-2, 2 mg/mL galactitol, 1.5 mM NAD^+^, and 20 mM Tris–HCl buffer (pH 7.0) was incubated at 30 °C and 200 rpm for 12 h. After the enzymatic reactions, reaction products were analyzed by GC/MS.

To measure the transglycosylase activity of crude cell-free lysate enzymes of strain EJ4 or D452-2, the reaction mixture containing 1 mg/mL crude cell-free lysate enzymes obtained from EJ4 or D452-2, 2 mg/mL of a sugar substrate such as glucose, galactose, cellobiose, or xylose, and 20 mM Tris–HCl buffer (pH 7.0) was incubated at 30 °C and 200 rpm for 12 h. After the enzymatic reactions, reaction products were analyzed by thin-layer chromatography (TLC).

### TLC analysis of enzymatic reaction products

Crude enzyme reaction products were analyzed by TLC on a silica gel plate (Sigma-Aldrich, St. Louis, MO). The plate was developed in a solution composed of n-butanol, ethanol, and water (3:1:1, v/v/v) for 1 h. Then, visualization of the plate was conducted by means of a solution consisting of 10% (v/v) sulfuric acid and 0.2% (w/v) 1,3-dihydroxynaphthalene (Sigma-Aldrich) in ethanol.

### High-performance liquid chromatography

During the fermentation of strain EJ4, consumption of sugars, namely glucose, galactose, xylose, or cellobiose, and production of ethanol were monitored by high-performance liquid chromatography (HPLC; Agilent Technologies, Waldbronn, Germany) equipped with a refractive index detector and a H^+^ column (Rezex ROA-Organic Acid; Phenomenex, Torrance, CA). The column and refractive index detector temperatures were set to 50 °C, and 0.005 N H_2_SO_4_ served as a mobile phase at a flow rate of 0.6 mL/min.

### Preparation of yeast metabolome samples

For intracellular metabolite analysis, the fast filtration sampling method was employed as previously described with a slight modification [41]. Briefly, 0.5 mL of a cell culture of *S. cerevisiae* EJ4 grown until the exponential phase was collected and vacuum-filtered on a vacuum manifold system (Vac-Man Laboratory Vacuum Manifold, Promega, Madison, WI) assembled with a nylon membrane filter (0.45 µm pore size, 13 mm diameter, Whatman, Piscataway, NJ) and a filter holder (Millipore, Billerica, MA). The filtered cell culture was then washed with 2.5 mL of distilled water at room temperature. The entire process for fast filtration was finished within 1 min. The filter membrane containing the washed cells was quickly mixed with 1 mL of a pre-chilled acetonitrile–water mixture (1:1, v/v) and 100 µL of glass beads.

After adding a metabolite extraction solvent and glass beads to the cell pellet obtained by the fast filtration, the extraction mixture was vortexed for 3 min to extract intracellular metabolites of *S. cerevisiae* by disruption of cell membrane. The extraction mixture was then centrifuged at 16,100×*g* for 3 min at 4 °C, and 0.8 mL of the supernatant containing the intracellular metabolites was dried in a speed vacuum concentrator for 6 h. For analysis of extracellular metabolites, 20 µL of the culture supernatant was dried in the speed vacuum concentrator for 6 h.

### Metabolite analysis by GC/MS

Prior to GC/MS analysis, the samples were derivatized by methoxyamination and trimethylsilylation. For methoxyamination, 10 µL of 40 mg/mL methoxyamine chloride in pyridine (Sigma-Aldrich) was added to the samples and incubated for 90 min at 30 °C and 200 rpm. The samples were then trimethylsilylated by adding 45 µL of *N*-methyl-*N*-trimethylsilyltrifluoroacetamide (Sigma-Aldrich) with incubation for 30 min at 37 °C and 200 rpm.

For GC/MS, the derivatized metabolite samples were applied to an Agilent 7890A GC/5975C MSD system (Agilent Technologies) equipped with an RTX-5Sil MS capillary column (30 m × 0.25 mm, 0.25 µm film thickness; Restek, Bellefonte, PA) and an additional 10-m-long integrated guard column. One microliter of the derivatized sample was injected into the GC inlet in splitless mode. The oven temperature was initially set to 150 °C for 1 min, after which the temperature was increased to 330 °C at 20 °C/min, where it was held for 5 min. The mass spectra were recorded in a scan range 85–500 *m/z* at electron impact of 70 eV, and the temperatures of the ion source and transfer line were 230 and 280 °C, respectively.

### Metabolite data processing and statistical analyses

The raw data obtained from GC/MS analysis were pre-processed in automated mass spectral deconvolution and identification system (AMDIS) software [42] for peak detection and deconvolution of mass spectra. The pre-processed data were uploaded into SpectConnect (http://spectconnect.mit.edu) [43] for peak alignment and for generating the data matrix with Golm Metabolome Database (GMD) mass spectral reference library [44]. The normalized abundance values for each metabolite were obtained by dividing peak intensity with dry cell weight. For statistical analysis, such as PCA and HCA presented as a heat map, Statistica (version 7.1; StatSoft, Palo Alto, CA) and MultiExperiment Viewer software [45] were used, respectively.

### Alkaline phosphatase assay

The autophagy magnitude of strain EJ4 grown on different carbon sources (glucose, galactose, xylose, or cellobiose) was measured as previously described [46]. Pho8∆60 was integrated into the EJ4 strain for alkaline phosphatase (ALP) activity measurement. Next, 4–5 OD_600_ units of yeast cells were collected in the exponential phase and washed twice with cold distilled water. The cells were then resuspended in 500 µL of ice-cold assay buffer (250 mM Tris–HCl at pH 9.0, 10 mM MgSO_4_, 10 mM ZnSO_4_) and disrupted by means of a Fast Prep-24 homogenizer (MP Biomedicals, Solon, OH) with glass beads. After centrifugation at 21,130×*g* at 4 °C for 10 min, 10 µL of the supernatant was added to 100 µL of assay buffer, and 10 µL of 55 mM α-naphthyl phosphate was then added to start the reaction. The fluorescence intensity of emission at 472 nm after excitation at 345 nm was measured at 2-min intervals on a microplate reader for 20 min. Protein concentration was determined with the Pierce BCA Protein Assay Kit. The ALP activity was calculated as emission per unit of the protein amount in the reaction (mg) and reaction time (min). The relative ALP activity for different carbon sources was normalized to the activity during the growth on glucose.

## Additional files

**Additional file 1: Table S1.** A total of 73 intracellular metabolites of strain EJ4, as identified by GC/MS analysis.

**Additional file 2: Table S2.** Twenty metabolites with high absolute loadings on PC1 and PC2, as determined by PCA.

**Additional file 3: Figure S1.** Accumulation of galactitol and tagatose during galactose fermentation by strain EJ4. Final concentrations of galactitol and tagatose in the culture supernatant of EJ4 were measured by HPLC after galactose depletion at 33 h.

**Additional file 4: Figure S2.** GC/MS analysis of the reaction products obtained from in vitro reactions with galactitol using a crude enzyme extract of *S. cerevisiae* D452-2 or EJ4 to measure galactitol 2-dehydrogenase acitivity. The enzyme mixture containing 1 mg/mL crude cell-free lysate enzymes obtained from *S. cerevisiae* strain D452-2 or EJ4, 2 mg/mL galactitol, 1.5 mM NAD^+^, and 20 mM Tris–HCl buffer (pH 7.0) was incubated at 30 °C and 200 rpm for 12 h.

**Additional file 5: Figure S3.** TLC analysis of the reaction products obtained from in vitro reactions with various substrates, namely galactose (Gal), cellobiose (CB), xylose (Xyl), and glucose (Glc) using a crude enzyme extract of *S. cerevisiae* D452-2 or EJ4 to measure transglycosylase activity. The enzyme mixture containing 1 mg/mL crude cell-free lysate enzymes obtained from *S. cerevisiae* D452-2 or EJ4, 2 mg/mL one of the substrates, and 20 mM Tris–HCl buffer (pH 7.0) was incubated at 30 °C and 200 rpm for 12 h. The products of the enzymatic reactions involving crude cell-free lysate enzymes of (**A**) *S. cerevisiae* D452-2 or (**B**) EJ4 were analyzed by TLC.

**Additional file 6: Figure S4.** Relative ALP activity of strain EJ4 grown on different carbon sources, glucose, galactose, xylose, or cellobiose. Yeast cells were harvested in the exponential phase for measurement of the ALP enzymatic activity. Glucose depletion: yeast cells were cultured in the presence of glucose, and samples were taken when all the glucose was consumed.

## Abbreviations

XR: xylose reductase
XDH: xylitol dehydrogenase
GC/MS: gas chromatography/mass spectrometry
PCA: principal component analysis
HCA: hierarchical cluster analysis
PP: pentose phosphate
GABA: γ-aminobutyric acid
TCA: tricarboxylic acid
