# Supplementary material for: Promiscuous activities of heterologous enzymes lead to unintended metabolic rerouting in Saccharomyces cerevisiae engineered to assimilate various sugars from renewable biomass
Source: Biotechnol Biofuels. 2018 May 14;11:140. doi: 10.1186/s13068-018-1135-7 (PMC5950193; doi:10.1186/s13068-018-1135-7)
Supplement: Supplementary file 1 — Additional file 1: Table S1. A total of 73 intracellular metabolites of strain EJ4, as identified by GC/MS analysis. [file 13068_2018_1135_MOESM1_ESM.doc]

**Additional file 1**

**Table S1** A total of 73 intracellular metabolites of strain EJ4, as identified by GC-MS analysis

| **Amino acids (20)** |  |
| --- | --- |
| Alanine  Arginine  Asparagine  Aspartate  Glutamate  Glutamine  Glycine  Histidine  Homocysteine  Inosine | Isoleucine  Norleucine  Ornithine  Phenylalanine  Proline  Serine  Threonine  Tryptophan  Tyrosine  Valine |
| **Sugars (12)** |  |
| Cellobiose  Galactitol  Galactose  Glucose  Lactitol  Mannitol  **Phosphates (9)** | Tagatose  Trehalose  Xylitol  Xylobiose  Xylose  Xylulose |
| Adenosine 5-monophosphate  Fructose 1,6-bisphosphate  Gluconic acid 6-phosphate  Glycerol 3-phosphate  Myo-Inositol 2-phosphate | Phosphate  Pyrophosphate  Sorbitol 6-phosphate  Xylulose 5-phosphate |
| **Fatty acids (5)** |  |
| 1-Octadecanol  2-Hexadecenoic acid  Hexadecanoic acid | Octadecanoic acid  Tetradecanoic acid |
| **Organic acids (15)** |  |
| 1-Pyrroline-3-hydroxy-5-carboxylate  2-Amino-butanoic acid  4-Amino-butanoic acid  4-Hydroxyphenylacetate  2-Hydroxyglutarate  5-Aminolevulinic acid  Aminomalonate  3-Phosphoglycerate | 4,8-Dihydroxy-quinoline-2-carboxylate  Benzoate  Citrate  Fumarate  Furan-2-carboxylic acid  Kynurenic acid  *N*-acetyl-glutamate |
| **Nucleosides (2)** |  |
| Adenine | Methylthioadenosine |
| **Others (10)** |  |
| 2-(4-Hydroxyphenyl)ethanol  3-Hydroxypyridine  Hydroxylamine  Hypoxanthine  Ethanolamine | Tetracosane  Uracil  Glucono-1,5-lactone  Glycerol  Ornithine-1,5-lactam |
