# Supplementary material for: Promiscuous activities of heterologous enzymes lead to unintended metabolic rerouting in Saccharomyces cerevisiae engineered to assimilate various sugars from renewable biomass
Source: Biotechnol Biofuels. 2018 May 14;11:140. doi: 10.1186/s13068-018-1135-7 (PMC5950193; doi:10.1186/s13068-018-1135-7)
Supplement: Supplementary file 2 — Additional file 2: Table S2. Twenty metabolites with high absolute loadings on PC1 and PC2, as determined by PCA. [file 13068_2018_1135_MOESM2_ESM.doc]

**Additional file 2**

**Table S2 Twenty metabolites with high absolute loadings on PC1 and PC2, as determined by PCA**

| **PC1** |  | **PC2** |  |
| --- | --- | --- | --- |
| **Metabolite** | **Loading** | **Metabolite** | **Loading** |
| 2-(4-Hydroxyphenyl)ethanol | 0.8519 | Tryptophan | 0.8275 |
| Xylose | 0.8293 | Phenylalanine | 0.7007 |
| Furan-2-carboxylic acid | 0.8284 | Lactitol | 0.6782 |
| Xylitol | 0.7921 | Galactitol | 0.6588 |
| Kynurenic acid | 0.7913 | 2-Hexadecenoic acid | 0.6493 |
| Inosine | 0.7710 | Tagatose | 0.6463 |
| Mannitol | 0.7707 | Sorbitol-6-phosphate | 0.6369 |
| Trehalose | 0.7046 | Galactose | 0.6309 |
| Xylobiose | 0.6948 | Isoleucine | 0.6181 |
| Octadecanoic acid | 0.6937 | Glycine | 0.6079 |
| Hypoxanthine | 0.6868 | Asparagine | − 0.8441 |
| Ornithine | − 0.8296 | Cellobiose | − 0.8201 |
| 5-Aminolevulinic acid | − 0.7960 | Glucono-1,5-lactone | − 0.7838 |
| Adenosine 5-monophosphate | − 0.7884 | Citrate | − 0.7221 |
| Aspartate | − 0.7815 | Glutamine | − 0.6841 |
| Pyrophosphate | − 0.7750 | 4-Hydroxyphenylacetate | − 0.6593 |
| Aminomalonate | − 0.7673 | 4-Amino-butanoic acid | − 0.6061 |
| Threonine | − 0.7290 | Homocysteine | − 0.5964 |
| Glutamate | − 0.6956 | Alanine | − 0.5907 |
| Adenine | − 0.6685 | 2-Amino-butanoic acid | − 0.5907 |
