# Supplementary material for: Promiscuous activities of heterologous enzymes lead to unintended metabolic rerouting in Saccharomyces cerevisiae engineered to assimilate various sugars from renewable biomass
Source: Biotechnol Biofuels. 2018 May 14;11:140. doi: 10.1186/s13068-018-1135-7 (PMC5950193; doi:10.1186/s13068-018-1135-7)
Supplement: Supplementary file 3 — Additional file 3: Figure S1. Accumulation of galactitol and tagatose during galactose fermentation by strain EJ4. Final concentrations of galactitol and tagatose in the culture supernatant of EJ4 were measured by HPLC after galactose depletion at 33 h. [file 13068_2018_1135_MOESM3_ESM.doc]

**Additional file 3**


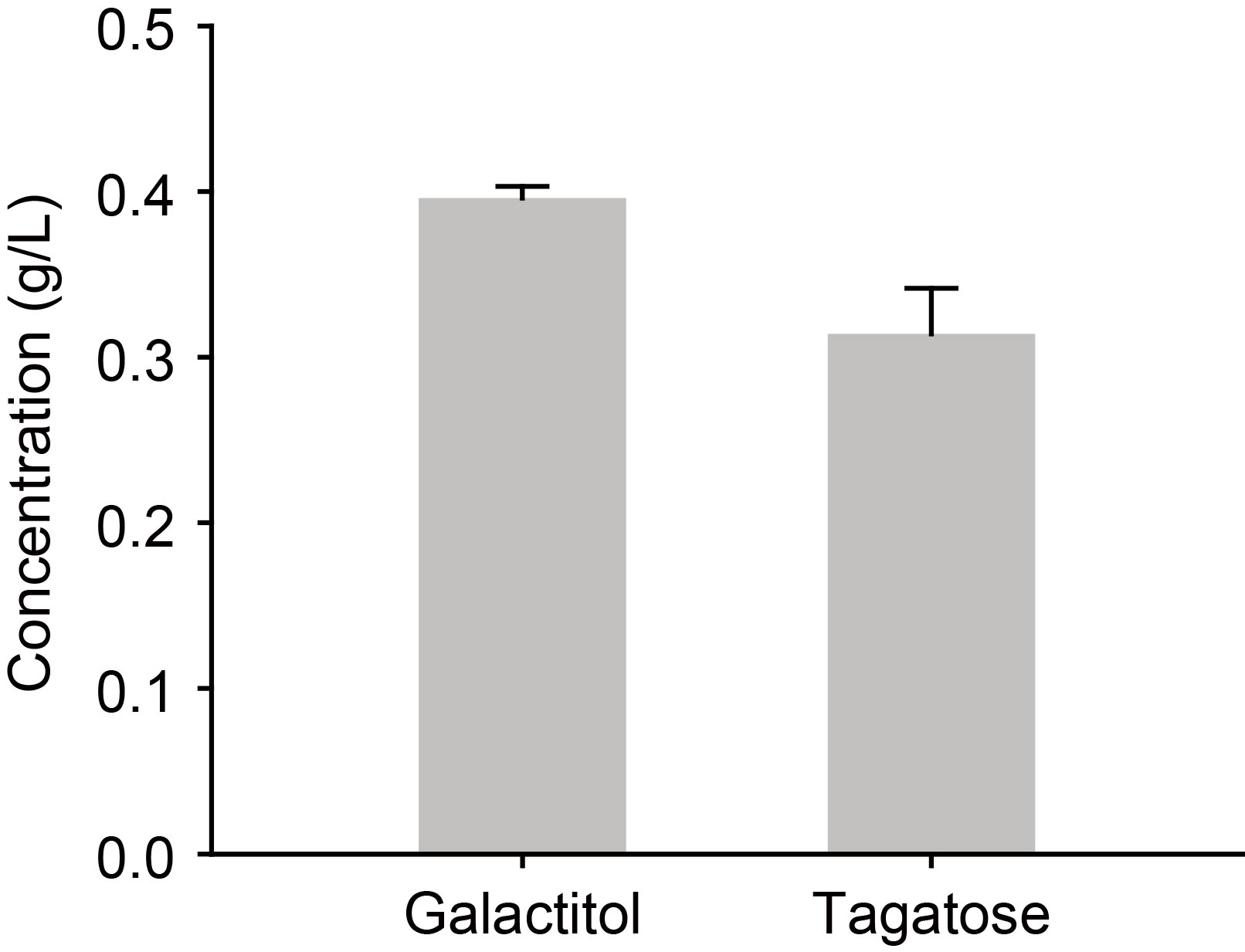


**Figure S1** Accumulation of galactitol and tagatose during galactose fermentation by strain EJ4. Final concentrations of galactitol and tagatose in the culture supernatant of EJ4 were measured by HPLC after galactose depletion at 33 h
