# Supplementary material for: Promiscuous activities of heterologous enzymes lead to unintended metabolic rerouting in Saccharomyces cerevisiae engineered to assimilate various sugars from renewable biomass
Source: Biotechnol Biofuels. 2018 May 14;11:140. doi: 10.1186/s13068-018-1135-7 (PMC5950193; doi:10.1186/s13068-018-1135-7)
Supplement: Supplementary file 4 — Additional file 4: Figure S2. GC/MS analysis of the reaction products obtained from in vitro reactions with galactitol using a crude enzyme extract of S. cerevisiae D452-2 or EJ4 to measure galactitol 2-dehydrogenase acitivity. The enzyme mixture containing 1 mg/mL crude cell-free lysate enzymes obtained from S. cerevisiae strain D452-2 or EJ4, 2 mg/mL galactitol, 1.5 mM NAD+, and 20 mM Tris–HCl buffer (pH 7.0) was incubated at 30 °C and 200 rpm for 12 h. [file 13068_2018_1135_MOESM4_ESM.doc]

**Additional file 4**


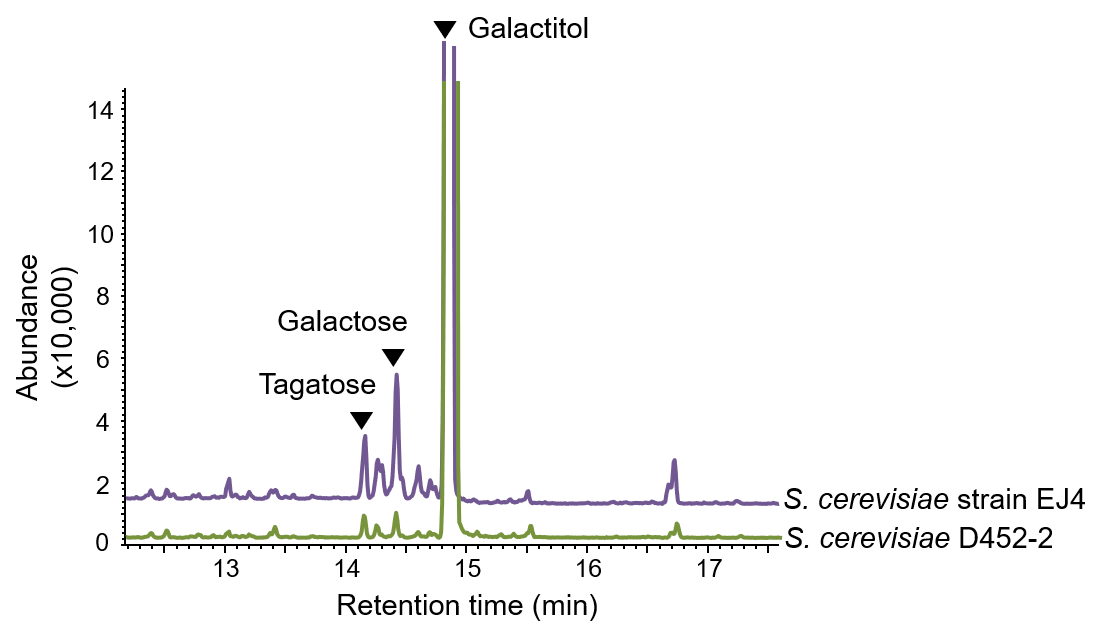


**Figure S2** GC/MS analysis of the reaction products obtained from *in vitro* assay of xylulose reductase (encoded by *XYL2*) on galactitol. The enzyme mixture containing 1 mg/mL crude cell-free lysate enzymes obtained from *S. cerevisiae* strain D452-2 or EJ4, 2 mg/mL xylulose, 1.5 mM NADH, and 20 mM Tris-HCl buffer (pH 7.0) was incubated at 30°C and 200 rpm for 12 h
