# Supplementary material for: Promiscuous activities of heterologous enzymes lead to unintended metabolic rerouting in Saccharomyces cerevisiae engineered to assimilate various sugars from renewable biomass
Source: Biotechnol Biofuels. 2018 May 14;11:140. doi: 10.1186/s13068-018-1135-7 (PMC5950193; doi:10.1186/s13068-018-1135-7)
Supplement: Supplementary file 5 — Additional file 5: Figure S3. TLC analysis of the reaction products obtained from in vitro reactions with various substrates, namely galactose (Gal), cellobiose (CB), xylose (Xyl), and glucose (Glc) using a crude enzyme extract of S. cerevisiae D452-2 or EJ4 to measure transglycosylase activity. The enzyme mixture containing 1 mg/mL crude cell-free lysate enzymes obtained from S. cerevisiae D452-2 or EJ4, 2 mg/mL one of the substrates, and 20 mM Tris–HCl buffer (pH 7.0) was incubated at 30 °C and 200 rpm for 12 h. The products of the enzymatic reactions involving crude cell-free lysate enzymes of (A) S. cerevisiae D452-2 or (B) EJ4 were analyzed by TLC. [file 13068_2018_1135_MOESM5_ESM.doc]

**Additional file 5**


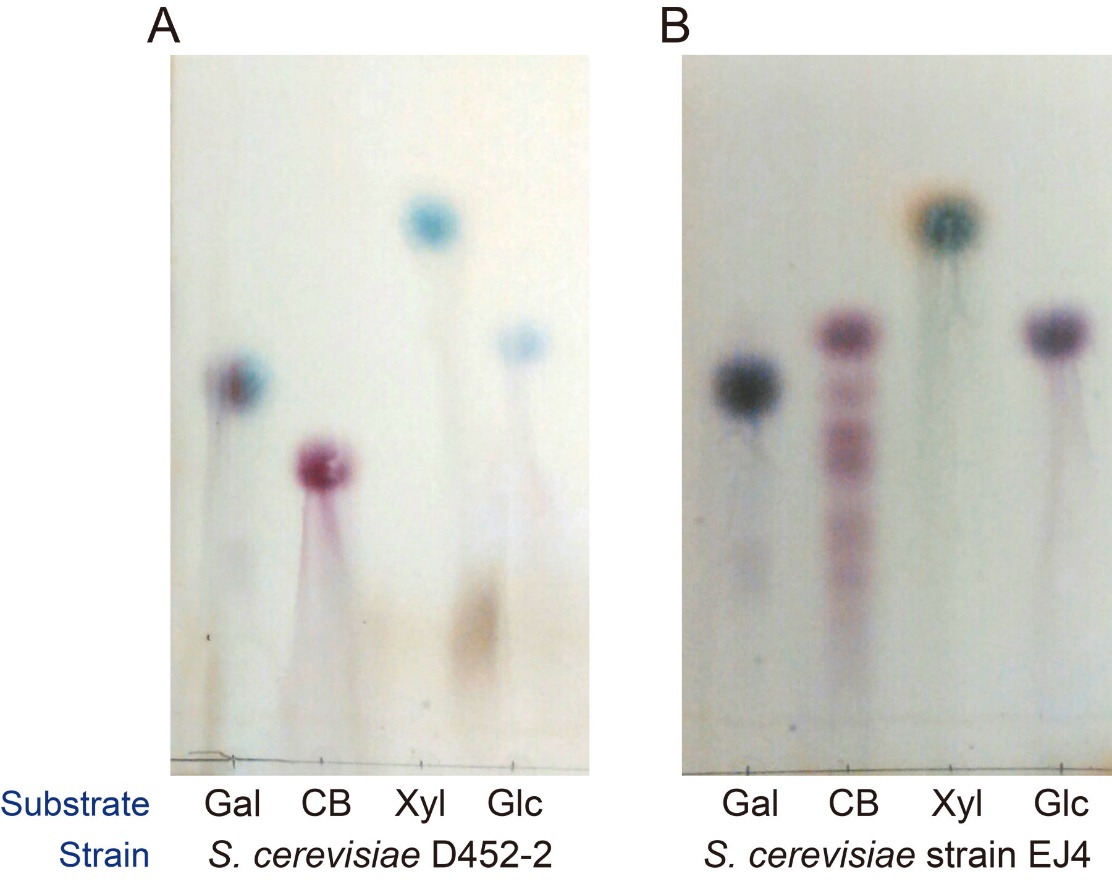


**Figure S3** TLC analysis of the reaction products obtained from *in vitro* activity assays of transglycosylase with various substrates, namely galactose (Gal), cellobiose (CB), xylose (Xyl), and glucose (Glc). The enzyme mixture containing 1 mg/mL crude cell-free lysate enzymes obtained from *S. cerevisiae* D452-2 or EJ4, 2 mg/mL one of the substrates, and 20 mM Tris-HCl buffer (pH 7.0) was incubated at 30°C and 200 rpm for 12 h. The products of the enzymatic reactions involving crude cell-free lysate enzymes of (**A**) *S. cerevisiae* D452-2 or (**B**) EJ4 were analysed by TLC
