# Supplementary material for: Promiscuous activities of heterologous enzymes lead to unintended metabolic rerouting in Saccharomyces cerevisiae engineered to assimilate various sugars from renewable biomass
Source: Biotechnol Biofuels. 2018 May 14;11:140. doi: 10.1186/s13068-018-1135-7 (PMC5950193; doi:10.1186/s13068-018-1135-7)
Supplement: Supplementary file 6 — Additional file 6: Figure S4. Relative ALP activity of strain EJ4 grown on different carbon sources, glucose, galactose, xylose, or cellobiose. Yeast cells were harvested in the exponential phase for measurement of the ALP enzymatic activity. Glucose depletion: yeast cells were cultured in the presence of glucose, and samples were taken when all the glucose was consumed. [file 13068_2018_1135_MOESM6_ESM.doc]

**Additional file 6**

**Figure S4** Relative ALP activity of strain EJ4 grown on different carbon sources, glucose, galactose, xylose, or cellobiose. Yeast cells were harvested in the exponential phase for measurement of the ALP enzymatic activity. Glucose depletion: yeast cells were cultured in the presence of glucose, and samples were taken when all the glucose was consumed
